# Genome Size and Chromosome Number Evolution in Korean *Iris* L. Species (Iridaceae Juss.)

**DOI:** 10.3390/plants9101284

**Published:** 2020-09-28

**Authors:** Bokyung Choi, Hanna Weiss-Schneeweiss, Eva M. Temsch, Soonku So, Hyeon-Ho Myeong, Tae-Soo Jang

**Affiliations:** 1 Department of Biological Science, College of Bioscience and Biotechnology, Chungnam National University, Daejeon 34134, Korea; cbokyung@cnu.ac.kr; 2 Department of Botany and Biodiversity Research, University of Vienna, Rennweg 14, A-1030 Vienna, Austria; hanna.schneeweiss@univie.ac.at (H.W.-S.); eva.temsch@univie.ac.at (E.M.T.); 3 Korea National Park Research Institute, 171, Dangu-ro, Wonju-si 26441, Gangwon-do, Korea; ssk822@knps.or.kr (S.S.); ecomyung@knps.or.kr (H.-H.M.)

**Keywords:** chromosome number, genome size, *Iris*, karyotype, systematics

## Abstract

Chromosome numbers, karyotypes, and genome sizes of 14 *Iris* L. (Iridaceae Juss.) species in Korea and their closely related taxon, *Sisyrinchium rosulatum*, are presented and analyzed in a phylogenetic framework. To date, understanding the chromosomal evolution of Korean irises has been hampered by their high chromosome numbers. Here, we report analyses of chromosome numbers and karyotypes obtained via classic Feulgen staining and genome sizes measured using flow cytometry in Korean irises. More than a two-fold variation in chromosome numbers (2*n* = 22 to 2*n* = 50) and over a three-fold genome size variation (2.39 pg to 7.86 pg/1 C) suggest the putative polyploid and/or dysploid origin of some taxa. Our study demonstrates that the patterns of genome size variation and chromosome number changes in Korean irises do not correlate with the phylogenetic relationships and could have been affected by different evolutionary processes involving polyploidy or dysploidy. This study presents the first comprehensive chromosomal and genome size data for Korean *Iris* species. Further studies involving molecular cytogenetic and phylogenomic analyses are needed to interpret the mechanisms involved in the origin of chromosomal variation in the *Iris*.

## 1. Introduction

Chromosomal changes play a major role in plant evolution and are thus important in diversification and speciation in angiosperms [1,2,3]. Variation of chromosome numbers, ploidy levels, and genome sizes have been frequently analyzed for a better understanding of evolutionary patterns and species relationships in plants [4,5,6,7]. The genome size values, in combination with data on chromosome numbers, allow ploidy levels to be inferred and can also provide insights into evolutionary relationships between closely related taxa in wild plant groups [7,8,9,10,11,12]. While the monocots are known to have the widest range of genome sizes (0.2–152.2 pg/1 C) among angiosperms [13,14,15], the known genome sizes in the family Iridaceae range from 0.5 pg/1 C in *Hesperantha* and *Sisyrinchium* species to 31.4 pg/1 C in *Moraea* taxa [15,16].

Among monocots, *Iris* is an excellent system in which to study the evolutionary patterns of chromosome number and genome size evolution because this group has an exceptionally high diversity of chromosome numbers (*n* = 7, 8, 9, 10, 11, 12, 13, 15, 16, 17, 18, 19, 20, 21, 22, 24, 25, 27, 36, 54) [17] and genome sizes (1.05–28.2 pg/1 C) [15]. The genus *Iris* L. is perennial and comprises approximately 300 species worldwide, with the greatest number of endemic species occurring in the Mediterranean and Asia [18,19,20,21]. Generic and sub-generic classification of the genus *Iris* has been mainly based on morphological characters of flower organs and roots [22,23]. Subsequently, Wilson [24] recognized nine subgenera in the genus based on phylogenetic analysis of chloroplast sequence data: *Iris*, *Crossiris* Spach, *Limniris*, *Lophiris* (Tausch) C. A. Wilson, *Nepalensis*, *Pardanthopsis* (Hance) Baker, *Siphonostylis* (W. Schulze) C. A. Wilson, *Xiphium*, and *Xyridon* (Tausch) Spach.

Currently, 14 species from *Iris* (subgenus *Limniris* and *Pardanthopsis*) are recognized in Korea: *Iris ensata* Thunb., *I. dichotoma* Pall., *I. domestica* (L.) Goldblatt & Mabb., *I. koreana* Nakai, *I. oxypetala* Bunge, *I. pseudacorus* L., *I. uniflora* Pall. ex Link, *I. laevigata* Fisch., *I. sanguinea* Donn ex Hornem., *I. setosa* Pall. ex Link, *I. odaesanensis* Y.N. Lee, *I. minutoaurea* Makino, *I. rossii* Baker, *I. ruthenica* Ker Gawl. [24,25,26,27]. Species of the Korean irises usually have a narrow geographical distribution, and some are endemic (*Iris koreana*) or subendemic to Korea (*Iris odaesanensis* with a small/disjunct population in Jilin Province, China) [19,25]. While several species of Korean irises (*I. dichotoma*, *I. laevigata*, *I. setosa*, and *I. ruthenica*) are either endangered or threatened due to habitat loss caused by anthropogenic activities [25,28], *Iris pseudacorus* and *Sisyrinchium rosulatum* were introduced as horticultural elements and are now naturalized [25]. Despite an increasing importance of conservation status of the Korean irises, comprehensive systematic studies including morphological, phylogenetic, and cytological analyzes have not been performed in the group. Molecular phylogenetic analyses based on plastid and nuclear ribosomal DNA data have allowed for the inferring established relationship patterns of major lineages of among the Korean irises [27,29,30], although some sectional and species level relationships still remain unresolved.

Worldwide, chromosome numbers in the genus *Iris* have been reported for 168 taxa in validly published references (Table S1; Chromosome Counts Database, CCDB, ver. 145, http://ccdb.tau.ac.il/Angiosperms/Iridaceae/Iris/) [17]. Chromosome numbers and karyotypes have been reported for eight species of the Korean irises (*I. ensata*, *I. dichotoma*, *I. koreana*, *I. oxypetala*, *I. pseudacorus*, *I. uniflora*, *I. setosa*, *I. sanguinea*), but none of the studied plants were collected from natural populations [31]. Thus, detailed cytogenetic studies of Korean irises from natural populations are required. Analyses of chromosome numbers accompanied by genome size measurements allow for inferences of the incidence of polyploidy [5,7]. Although the genome size data of 44 *Iris* species have been conducted so far [15], the nuclear DNA amounts using flow cytometry for Korean irises are not available to date [15].

Thus, this study examines the patterns of chromosome number evolution, karyotypes, and genome size variation in a phylogenetic context in the Korean irises. The specific aims of this study are (1) to determine chromosome numbers and size variation as well as karyotypes structure all 14 Korean *Iris* species from multiple populations; (2) to examine patterns of genome size change in this group of taxa to address to the lack of the C-value data in the genus *Iris*; and (3) to interpret the patterns of evolution of cytological data within the available phylogenetic framework to better understand the genome evolution in Korean irises.

## 2. Materials and Methods

### 2.1. Plant Materials

A total of 76 individuals representing 36 populations of 14 species of *Iris* and one outgroup species *Sisyrinchium rosulatum* were collected from natural populations with few individuals used from other sources (cultivation; Table 1). The plants used in this study were transplanted in a glasshouse in Chungnam National University, Daejeon, Republic of Korea. All individuals were used for analyses of chromosome numbers, karyotypes and measurements of genome size (Table 1). Voucher specimens of the examined plants were deposited in CNUK (Chungnam National University Herbarium). Four Korean *Iris* species (*I. dichotoma*, *I. laevigata*, *I. setosa*, *I. ruthenica*) that are endangered and threatened were collected with special permissions from the Korean Government (collected under permits no. 2018-18, 2019-13, 2019-14, 2019-20, and 2019-30; Table 1). In order to avoid illegal removal to the endangered or vulnerable species, we refrain from providing details of the locality information (GPS coordinates) in this study.

### 2.2. Chromosome Numbers and Karyotype Analysis

Fresh root tips were pretreated in 0.05% colchicine (Sigma-Aldrich Co., St. Louis, USA) solution at room temperature in the dark for 4.5 h. The sampled roots were fixed in ethanol:acetic acid (3:1) for two hours at room temperature and then stored at −20 °C. For karyotype and chromosome number analyses, meristematic root cells were used (Table 1). Prior to staining, the fixed roots were washed with tap water, and hydrolyzed in 5 N HCL at room temperature for 30 min. Subsequently, to perform Feulgen staining, the root tips were treated in Schiff′s reagent (Merck KGaA, Darmstadt, Germany) in darkness for 1 h. Microscopic slides were prepared by squashing the meristematic tissue in 60% acetic acid. A minimum of three well spread chromosome plates were selected for the analyses [32]. The chromosomes were cut using Corel Photo-Paint 12.0 (Corel Company, Ottawa, Canada). For chromosomal measurements, MicroMeasure ver.3.3 program (https://micromeasure.software.informer.com/3.3/) [33] was used, and a minimum of three chromosomal spreads were measured with a comparable medium degree of chromosomal condensation (Table 2).

### 2.3. Genome Size Measurement by Flow Cytometry (FCM)

Genome sizes of 76 individuals of 14 *Iris* species and *Sisyrinchium rosulatum* were measured using flow cytometry with *Solanum pseudocapsicum* (1 C = 1.29 pg) [34] or *Pisum sativum* “Kleine Rheinländerin” (1 C = 4.42 pg) [35] as an internal standard. Approximately 20–30 mg of fresh leaves of each sample were chopped with an internal reference standard using Otto′s buffer I [36]. Subsequently, each sample was filtered using a nylon mesh, and incubated with RNase A in a water-bath at 37 °C for 30 min. The nuclei staining was conducted using propidium iodide, which contains Otto´s buffer II, and the sample was stored at 4 °C. A total of three measurements of DNA content were estimated for each sample using a flow cytometer (Partec, Münster, Germany). 1 C-values were calculated using estimation of linear fluorescence intensity of nuclei of the examined taxon and internal standard. The coefficient of variation (CV) of all estimations were mostly below 3% on average, and never exceeded 7% [7].

## 3. Results and Discussion

### 3.1. Chromosome Numbers and Karyotypes of Iris L.

**Figure 1:**
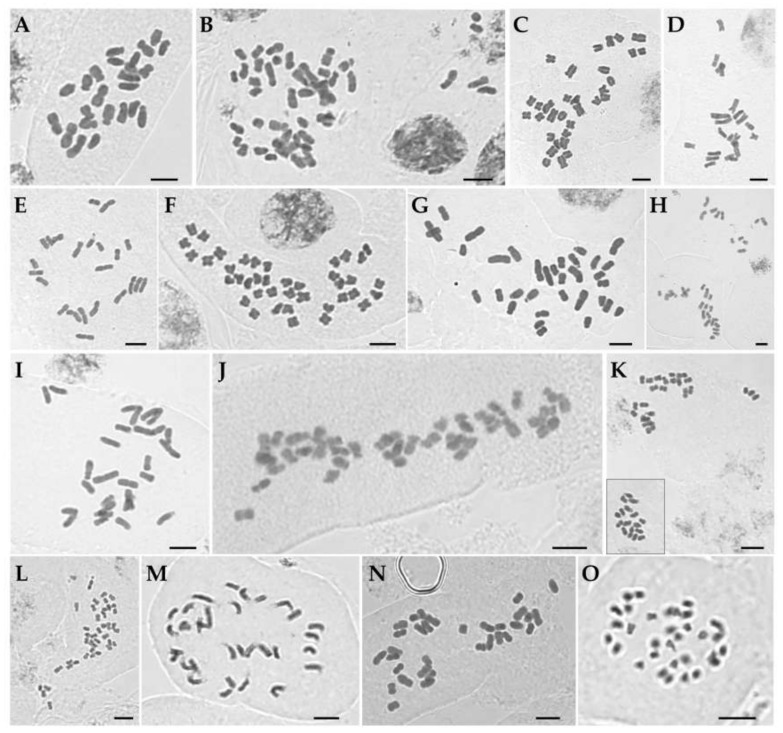
Mitotic metaphase chromosomes of Korean irises. (**A**) *Iris ensata* (2*n* = 24). (**B**) *I. koreana* (2*n* = 50). (**C**) *I. laevigata* (2*n* = 32). (**D**) *I. minutoaurea* (2*n* = 22). (**E**) *I. odaesanensis* (2*n* = 28). (**F**) *I. oxypetala* (2*n* = 40). (**G**) *I. pseudacorus* (2*n* = 34). (**H**) *I. rossii* (2*n* = 32). (**I**) *I. sanguinea* (2*n* = 28). (**J**) *I. setosa* (2*n* = 38). (**K**) *I. ruthenica* (2*n* = 42). **(L)** *I. uniflora* (2*n* = 42). (**M**) *I.* *dichotoma* (2*n* = 32). (**N**) *I. domestica* (2*n* = 32). (**O**) *Sisyrinchium rosulatum* (2*n* = 32). Inset in (**K**) shows same chromosome group of a single cell which were lying at some distance using high magnification objectives. Scale bar = 5 µm.

**Figure 2:**
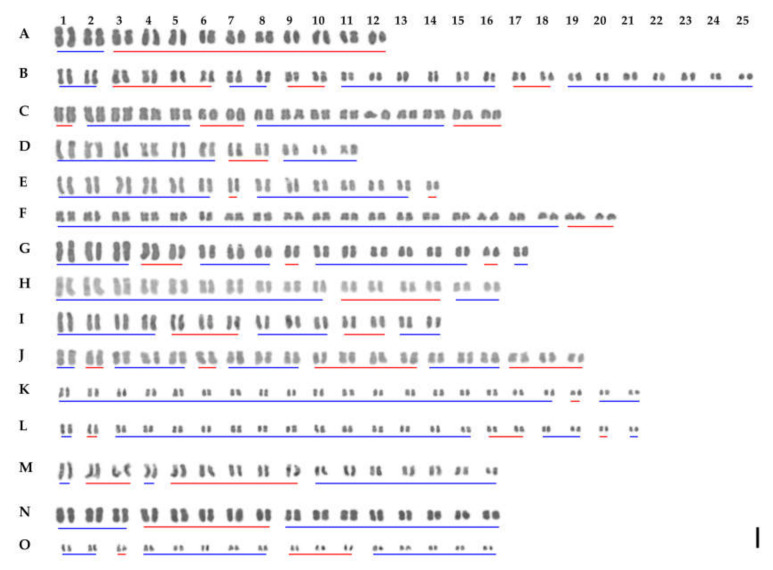
Karyotypes of the Korean irises. (**A**) *Iris ensata* (2*n* = 24). (**B**) *I. koreana* (2*n* = 50). (**C**) *I. laevigata* (2*n* = 32). (**D**) *I. minutoaurea* (2*n* = 22). (**E**) *I. odaesanensis* (2*n* = 28). (**F**) *I. oxypetala* (2*n* = 40). (**G**) *I. pseudacorus* (2*n* = 34). (**H**) *I. rossii* (2*n* = 32). (**I**) *I. sanguinea* (2*n* = 28). (**J**) *I. setosa* (2*n* = 38). (**K**) *I. ruthenica* (2*n* = 42). (**L**) *I. uniflora* (2*n* = 42). (**M**) *I. dichotoma* (2*n* = 32). (**N**) *I. domestica* (2*n* = 32). (**O**) *Sisyrinchium rosulatum* (2*n* = 32). Blue and red bars indicate metacentric and submetacentric chromosomes, respectively. Scale bar: 5 µm.

Chromosome numbers for 74 individuals of 14 currently recognized the Korean *Iris* species and two individuals of *Sisyrinchium rosulatum* are provided in Table 1 and Figure 1 and Figure 2. The karyotype analyses of each species with detailed chromosome size measurements are presented in Table 2 and Figure 2. The previously reported chromosome number for the genus *Iris* varied from 2*n* = 14 (*I. vorobievii*) to 2*n* = 108 (*I. versicolor*) representing approximately a 7.7-fold difference (Table S1). To date, the chromosome numbers have been determined for approximately 168 taxa of ca. 300 currently recognized species in the genus *Iris* worldwide (c. 56%; Table S1) [17].

#### 3.1.1. *Iris* L. Subgenus *Limniris* Spach Section *Limniris* Spach

The chromosome numbers in sect. *Limniris* ranged from 2*n* = 22 in *I. minutoaurea* to 2*n* = 50 in *I. koreana* (Table 1) confirming earlier studies [17]. *I. odaesanensis* (2*n* = 28) is reported here for the first time. Both analyzed plants of *I. ensata* had 2*n* = 24 (Figure 1A), AsI (asymmetry index) of 66.7% and karyotype formula of 2*n* = 4 m + 20 sm (Figure 2A) with Haploid Karyotype length (HKL) of 82.5 ± 1.7 µm. The two individuals of *I. koreana* had 2*n* = 50 (Figure 1B), with its AsI of 61.9% and the karyotype formula of 34 m + 16 sm (Figure 2B) and HKL of 141.7 ± 9.9 µm. The four plants of *I. laevigata* had 2*n* = 32 (Figure 1C). The species had AsI of 61.9%, karyotype formula of 2*n* = 22 m + 10 sm (Figure 2C) and HKL of 81.8 ± 1.7 µm. All 11 analyzed plants of *I. minutoaurea* had 2*n* = 22 (Figure 1D) and AsI of 58.3%. The karyotype formula was 18 m + 4 sm (Figure 2D) with HKL of 75.8 ± 2.4 µm. All eight plants of *I. odaesanensis* had 2*n* = 28 (Figure 1E) with AsI of 59.5%, karyotype formula of 2*n* = 24 m + 4 sm (Figure 2E) and HKL of 86.3 ± 2.4 µm. All four plants of *I. oxypetala* had 2*n* = 40 (Figure 2F), AsI of 59.1% and karyotype formula of 2*n* = 36 m + 4 sm (Figure 2F). HKL of this species was 86.4 ± 3.8 µm. All seven plants of *I. pseudacorus* had 2*n* = 34 (Figure 1G), AsI was 62.0% and the karyotype formula was 26 m + 8 sm (Figure 2G) with HKL of 107.1 ± 4.2 µm. All nine plants of *I. rossii* had 2*n* = 32 (Figure 1H). This species had AsI of 60.9%, karyotype formula of 2*n* = 24 m + 8 sm (Figure 2H) and HKL of 100.5 ± 3.0 µm. All seven plants of *I. sanguinea* possessed 2*n* = 28 (Figure 1I), AsI of 62.5%, and the karyotype formula of 18 m + 10 sm (Figure 2I) with HKL of 87.2 ± 2.8 µm. All five plants of *I. setosa* had 2*n* = 38 (Figure 1J), in agreement with previous reports [17] except for the one study reporting 2*n* = 40 for this species [31]. This deviating count might have potentially referred to another taxon (either a species or a variety), but it is difficult to verify due to unknown origin of the examined sample (e.g., cultivated material) [31]. Most previous studies used cultivated material. Thus, any discrepancies that might exist between different reports might have been affected by domestication and associated potential hybridization [31], as also suggested for Iberian irises [37]. *Iris setosa* had AsI of 61.6% and karyotype formula of 2*n* = 20 m + 18 sm (Figure 2J) with HKL of 122.9 ± 6.9 µm.

#### 3.1.2. *Iris* L. Subgenus *Limniris* Spach Section *Ioniris* Spach

All nine plants of *I. ruthenica* had 2*n* = 42 (Figure 1K), in agreement with previous reports (Table S1) [17]. This species had AsI of 58.0% and karyotype formula of 2*n* = 40 m + 2 sm (Figure 2K) with HKL of 78.3 ± 4.3 µm. The three analyzed plants of *I. uniflora* had 2*n* = 42 (Figure 1L) confirming previous reports (Table S1) [17]. Its AsI was 58.3% and karyotype formula of 2*n* = 34 m + 8 sm (Figure 2L) with HKL of 61.9 ± 1.9 µm.

#### 3.1.3. *Iris* L. Subgenus *Pardanthopis* (Hance) Baker

All four examined individuals of *I. dichotoma* had 2*n* = 32 (Figure 1M). This number was consistent with previous records for this species from Russian and Chinese populations [17], except for the study of Kim et al. [31], in which analyzed cultivated material was reported to have 2*n* = 34. This species had AsI of 61.9% and karyotype formula of 2*n* = 18 m + 14 sm (Figure 2M) with HKL of 97.0 ± 4.4 µm. *Iris domestica* had 2*n* = 32 (Figure 1N), which was in agreement with previous reports (Table S1) [17]. Its AsI was 59.7% and karyotype formula was 2*n* = 22 m + 10 sm (Figure 2N) with HKL of 92.8 ± 4.2 µm.

#### 3.1.4. *Sisyrinchium rosulatum* E.P.Bicknell.

Both of the *S. rosulatum* individuals analyzed had the chromosome number of 2*n* = 32 (Figure 1O), and the results were in agreement with previous reports [17]. The AsI of this species was 59.9% and karyotype formula was 2*n* = 24 m + 8 sm (Figure 2O) with HKL of 43.4 ± 1.9 µm.

### 3.2. Genome Size Variation in Iris L.

DNA content analyses using an internal standard revealed clear and well-defined peaks for all samples with the coefficients of variation (CV) for the internal standard and sample peaks ranging from 1.03% to 5.40% (mean ± S.D. = 2.24 ± 0.80) and from 1.23% to 6.50% (mean ± S.D. = 2.87 ± 1.06), respectively.

**Figure 3:**
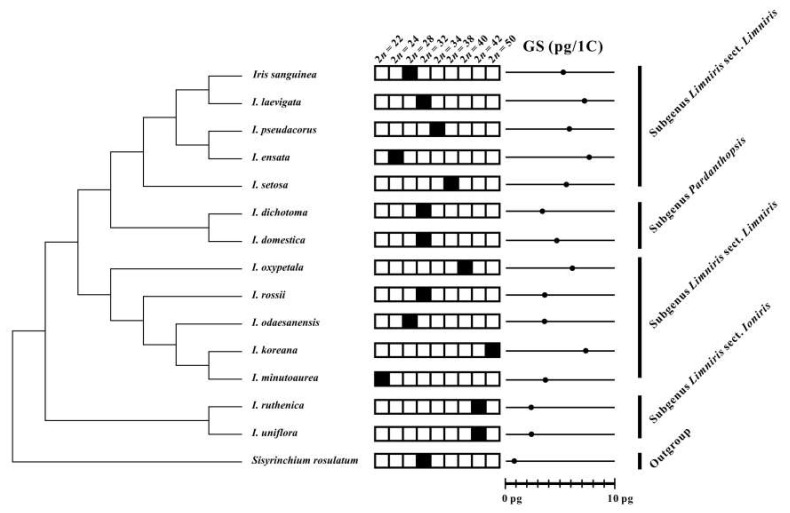
Chromosome numbers (2*n*; solid black squares) and genome sizes (GS) of Korean irises mapped on the phylogenetic tree based on nrITS sequences modified and simplified from Sim et al. [27]. The mean 1 C-values per species are indicated with solid dots.

Genome sizes for all Korean irises, except for *Iris pseudacorus*, are reported here for the first time (Table 1; Figure 3). The analysis revealed a 3.29-fold variation in nuclear DNA content among the analyzed taxa, with genome sizes ranging from 2.39 pg/1 C in *Iris ruthenica* to 7.86 pg/1 C in *I. ensata*. To date, the 1 C-values of 44 *Iris* species have been reported ranging from 1.05 pg/1 C in *I. versicolor* to 28.20 pg/1 C in *I. histrio* [15]. Genome size of *Iris pseudacorus* reported earlier (1 C = 5.67 pg) [38] is very similar with the current data (5.77–6.02 pg/1 C; Table 1). The outgroup species *Sisyrinchium rosulatum* had 0.90 pg/1 C (Table 1; Figure 3). The 1 C-values in Korean irises vary significantly among species, but are constant within populations and species, as previously observed in closely related species within a genus [10,39]. This suggests that ploidy levels in the populations of Korean irises examined in this study are at least partially stable within species. The new data presented here will allow for better understanding of the dynamics and patterns of chromosomal evolutionary changes in the genus *Iris* [15].

### 3.3. Evolution of Chromosome Numbers and Genome Sizes

**Figure 4:**
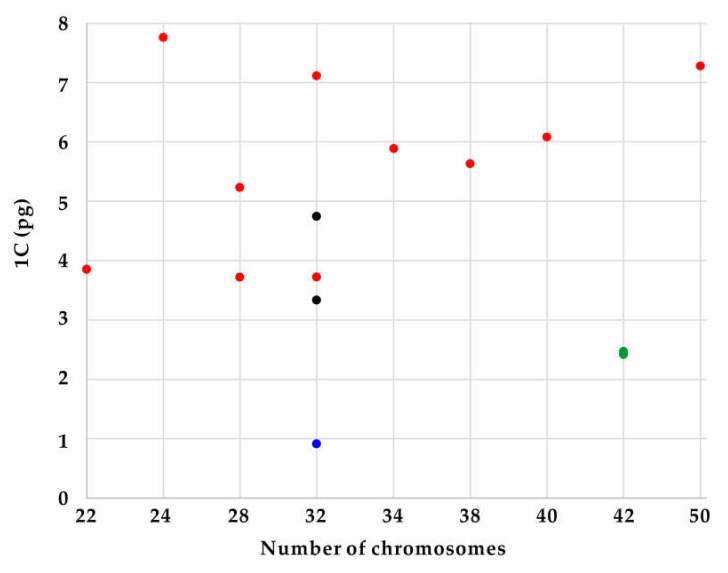
Distribution of genome size data [1 C (pg)] in relation to chromosome number (2*n*). Colors represent subgenus *Limniris* section *Limniris* (red), section *Ioniris* (green), subgenus *Pardanthopsis* (black), and *Sisyrinchium rosulatum* (blue).

The newly obtained chromosome numbers and genome sizes were mapped on nrITS phylogeny of Korean irises (modified from [27]). The data revealed that there is no correlation between chromosome numbers and genome sizes among the phylogenetic lineages of the analyzed Korean *Iris* species. As an example, *Iris ensata* with low chromosome number of 2*n* = 24 has the highest 1 C-value (7.76 pg/1 C) whereas its close relative *I*. *setosa* has 2*n* = 38, but 1.4-fold lower genome size (Figure 4). It suggests that dysploidy and polyploidy are not solely responsible for the observed variation of genome sizes and chromosome numbers, but also other processes, like independent accumulation or reduction of repetitive DNAs amount (e.g., satellite DNAs and/or transposable elements) play an important role, as previously reported in other plant groups [7,40,41,42,43].

Other lineages within the genus *Iris* might be more stable evolutionarily. Both *Iris ruthenica* and *I. uniflora* (subgenus *Limniris* section *Ioniris*) have the same chromosome numbers of 2*n* = 42 and similar genome sizes (ca. 2.4 pg/1 C). Thus, the current cytogenetic data support the monophyly of this section, as proposed based on phylogenetic analyses of plastid markers [44,45,46], but also suggest low levels of genome dynamics accompanying their evolution. *Iris dichotoma* and *I. domestica* of the subgenus *Pardanthopsis* have also been recovered in monophyletic clade in ITS- and plastid-based phylogenetic analyses [46,47]. However, although they share the same chromosome numbers of 2*n* = 32, they differ nearly a 1.5-fold in genome sizes. Such variation of genome sizes suggests independent accumulation/loss of repetitive DNAs in the absence of numerical chromosome changes as reported also in other monocot genera [7,40].

In contrast, allopolyploidy can be hypothesized for the origin of *Iris koreana* (2*n* = 50) from closely related *I. minutoaurea* (2*n* = 22) and *I. odaesanensis* (2*n* = 28) [30]. It is also supported from the additive genome size of this taxon. Allopolyploidy has been implied to be an important process in the early diversification of the whole family of Iridaceae [48,49]. Thus, further data employing GISH (genomic *in situ* hybridization) will allow testing the allopolyploid origin of this and other species in the section *Limniris*.

The subgenus *Limniris* section *Limniris* has previously been shown to be polyphyletic [27,30,44,45,46]. This study shows that there is also quite wide variation of chromosome numbers and genome sizes within the newly identified polyphyletic clades of this subgenus (Figure 3 and Figure 4). It suggests that the genome size and chromosome number evolved rather dynamically in those evolutionary lineages, as indeed also observed in other monocot genera [11,50,51].

## 4. Conclusions

The present study provides the first comprehensive analyses of chromosome numbers, karyotype features and genome size variation of Korean irises from natural populations. Despite the large variation of chromosome numbers (*n* = 11, 12, 14, 16, 17, 19, 20, 21, 25), some variation of genome sizes (2.42–7.76 pg/1 C) has been demonstrated. This variation is only partially phylogenetically structured. Numerical chromosomal changes, both polyploidy and dysploidy, seem to be accompanied to a different degree by the likely independent changes in the amounts of repetitive DNAs in different phylogenetic lineages. Various combinations of these processes acting on the genomes of closely related species create complex patterns of genome size and chromosome number variation observed in Korean irises. Further analyses are needed to elucidate how these processes affect the evolution of the genomes of individual species, not only of the *Iris* species in Korea but ideally in a broader phylogenetic context of whole subgenera/sections.

## Figures and Tables

**Table 1:** Information on the plant material used for the karyotype and genome size analyses of *Iris* L. in Korea.

Taxon; Collection Number	Locality; GPS Coordinate; Collector	Chromosome Number (2*n*)	Genome Size 1 C ± S.D. (pg)
*Iris*			
Subgenus *Limniris* section *Limniris*			
*Iris ensata* Thunb.			**7.76 ^4^**
IMHAE103	Yang-Yang, Kangwon; N38°05′44″, E128°39′15″, 154 m; TS, SK, CM	24	7.86 ± 0.022
IMHAE117	Pyeong-Chang, Kangwon; N37°41′34.03″, E128°45′25.79″, 889 m; TS, SK, CM	24	7.66 ± 0.011
*I. koreana* Nakai ^1, 2^			**7.29 ^4^**
sck00042	Byeonsanbando National Park Endangered Species Botanical Garden (Cult.); SS, RI, CB, SK	50	7.22 ± 0.068
sck00043	Byeonsanbando National Park Endangered Species Botanical Garden (Cult.); SS, RI, CB, SK	50	7.35 ± 0.029
*I. laevigata* Fisch. ^1^			**7.12 ^4^**
Jebi-2	Gangwon; TS, CB	32	7.06 ± 0.010
Jebi-3	Gangwon; TS, CB	32	7.18 ± 0.046
*I. minutoaurea* Makino			**3.85 ^4^**
Gasan_8	Gasan, Kyungsang; N36°02′41″, E128°34′12″, 733 m; TS, CB, HJ	22	3.92 ± 0.008
JCKCK193269	Gasan, Kyungsang; N36°02′35″, E128°34′36″, 675 m; TS, CB, SK, YM	22	3.71 ± 0.171
JCKCK193273-1	Gasan, Kyungsang; N36°02′26″, E128°34′26″, 779 m; TS, CB, SK, YM	22	3.71 ± 0.024
JCKCK193273-2	Gasan, Kyungsang; N36°02′26″, E128°34′26″, 779 m; TS, CB, SK, YM	22	3.72 ± 0.006
JC041913	Gasan, Kyungsang; N36°02′44″, E128°33′51″, 629 m; TS, YM	22	4.09 ± 0.042
JCKC1932211-2	Gasan, Kyungsang; N36°02′28″, E128°34′18″, 806 m; TS, CB, SK, YM	22	3.79 ± 0.019
JCKC1932211-3	Gasan, Kyungsang; N36°02′28″, E128°34′18″, 806 m; TS, CB, SK, YM	22	3.70 ± 0.003
*I. odaesanensis* Y.N.Lee ^1, 3^			**3.72 ^4^**
YSG18-66_1	Gangwon; YS	28	3.67 ± 0.012
YSG18-66_2	Gangwon; YS	28	3.68 ± 0.011
YSG18-66_4	Gangwon; YS	28	3.70 ± 0.019
BKC928	Kyungsang; CB, YC	28	3.79 ± 0.007
JCKC1932271	Kyungsang; TS, CB, KS, YM	28	3.76 ± 0.021
JC041904	Kyungsang; TS, YM	28	3.74 ± 0.042
JC041903	Kyungsang; TS, YM	28	3.67 ± 0.015
JC041940	Kyungsang; TS, YM	28	3.73 ± 0.018
*I. oxypetala* Bunge			**6.08 ^4^**
CC0621-50	Ga-Gokri, Chungnam; N37°01′21″, E126°34′30″, 4 m; CB, CM	40	6.38 ± 0.014
CC20190621-58	Ga-Gokri, Chungnam; N37°01′17″, E126°34′32″, 3 m; CB, CM	40	6.06 ± 0.010
CC20190621-64	Ga-Gokri, Chungnam; N37°01′24″, E126°34′26″, 4 m; CB, CM	40	6.00 ± 0.017
JCCMH025	Gang-Wha, Incheon; N37°45′37″, E126°14′29″, 80 m; TS, CB, CM	40	5.90 ± 0.005
*I. pseudacorus* L.			**5.89 ^4^**
Keumjeong-34	Mt. Keumjeong, Busan; N35°14′ 5.5″, E129°03′24.6″, 455 m; CB, YK, YC	34	5.82 ± 0.012
Chungdae_2	Sinseong-dong, Daejeon; N36°22′44.8″, E127°20′35.5″, 83 m; CB	34	5.87 ± 0.017
CC20190621-1	Is. Hwangdo, Chungnam; N36°35′58.2″, E126°22′42.5″, 14 m; CB	34	5.87 ± 0.024
CC20190621-48	Dangjin, Chungnam; N37°01′21″, E126°34′30″E, 4 m; CB, CM	34	5.98 ± 0.011
J20190616-1	Nogokjae, Kimchoen; N36°04′04″, E128°12′50″, 242 m; TS, KS, CB	34	6.02 ± 0.026
BKC910	Mt. Keumjeong, Busan; N35°14′06″, E129°03′28″, 459 m; CB, YK, YC	34	5.77 ± 0.009
CC20190621-57	Ga-Gokri, Chungnam; N37°01′21″, E126°34′30″, 4 m; CB, CM	34	5.91 ± 0.009
*I. rossii* Baker			**3.73 ^4^**
SJH1830	Mt. Cheon-Gwan, Jeon-Ra; N34°32′39.4″, E126°55′40.8″, 225 m; SJH	32	3.69 ± 0.014
Pusanuni_2	Mt. Keumjeong, Busan; N35°14′23.8″, E129°4′27.23″, 169 m; CB, YK	32	3.72 ± 0.005
J513	Mun-San, Kyung-Gi; N37°57′55″, E126°56′13″, 58 m; TS	32	3.85 ± 0.010
J514	Mun-San, Kyung-Gi; N37°57′55″, E126°56′11″, 74 m; TS	32	3.67 ± 0.013
Jeokseong_2	Mun-San, Kyung-Gi; N37°57′55″, E126°56′13″, 58 m; TS	32	3.66 ± 0.005
Keumjeong_8	Mt. Keumjeong, Busan; N35°14′23.8″ E129°4′27.23″, 169 m; CB, CM	32	3.79 ± 0.018
JCK05_7	Mt. Saeng-Aeng; N36°19′21.9″, E126°30′45.7″, 110 m; TS, CB, SK	32	3.74 ± 0.033
JCKC190512	Mojioreum, Jeju; N33°23′42″, E126°45′58″, 247 m; TS, CB, SK, CM	32	3.75 ± 0.003
SCK00021	Mt. Bong-Wha, Chungnam; N36°47′08″, E126°26′20″, 79 m; CB, SK	32	3.71 ± 0.003
*I. sanguinea* Hornem.			**5.23 ^4^**
K02-41	Mt. Hwa-Ak, Kyung-Gi; N38°00′16.60″, E127°52′51.93″, 1097 m; SK	28	5.22 ± 0.012
Hwaya_1	Mt. Hwa-Ya, Kyung-Gi; N36°56′24.5″, E127°16′46.8″, 162 m; TS	28	5.30 ± 0.009
K025-04-01	Yeon-Cheon, Gangwon; N38°09′97.95″, E127°11′28.64″, 129 m; SK	28	5.42 ± 0.021
K025-04-04	Yeon-Cheon, Gangwon; N38°09′97.95″, E127°11′28.64″, 129 m; SK	28	5.18 ± 0.022
Munsan_1	Mun-San, Kyung-Gi; N37°57′55″, E126°56′13″, 58 m; TS	28	5.12 ± 0.009
Cheonma-3	Mt. Cheon-Ma, Kyung-Gi; N37°41′24″, E127°24′36″, 157 m; YS, SJH	28	5.11 ± 0.011
JC041939	Dae-Gu, Kyungsang; N35°54′44″, E128°38′46″, 629 m; TS, YM	28	5.25 ± 0.020
*I. setosa* Pall. ex Link ^1^			**5.63 ^4^**
Ga-3	Gangwon; TS, CB	38	5.55 ± 0.012
Ga_4-1	Gangwon; TS, CB	38	5.64 ± 0.007
Ga-7	Gangwon; TS, CB	38	5.57 ± 0.004
Seo-2	Gangwon; TS, CB	38	5.78 ± 0.016
Ga-5	Gangwon; TS, CB	38	5.59 ± 0.035
Subgenus *Limniris* section *Ioniris*			
*I. ruthenica* Ker Gawl. ^1^			**2.42 ^4^**
Mo_2	Jeju; YS, SJH	42	2.45 ± 0.004
J1-1(SSK)	Incheon; SS	42	2.42 ± 0.002
BKC939	Busan; CB, YM	42	2.45 ± 0.006
JC532	Daegu; TS, CM	42	2.39 ± 0.001
JC352-1	Daegu; TS, CM	42	2.43 ± 0.006
JC534-1	Daegu; TS, CM	42	2.42 ± 0.017
SCK00032	Chungnam; SS, CB, SK	42	2.42 ± 0.001
BKC937	Busan; CB	42	2.40 ± 0.001
JCCMH004	Incheon; TS, CB, CM	42	2.43 ± 0.002
*I. uniflora* Pall. ex Link			**2.46 ^4^**
JCK2019-77	Mt. Sorak, Gangwon; N38°09′27″, E128°29′19″, 790 m; TS, CB, SK	42	2.48 ± 0.002
JCK2019-78	Mt. Sorak, Gangwon; N38°09′27″, E128°29′19″, 790 m; TS, CB, SK	42	2.44 ± 0.003
JCK2019-79	Mt. Sorak, Gangwon; N38°09′27″, E128°29′19″, 790 m; TS, CB, SK	42	2.46 ± 0.003
Subgenus *Pardanthopsis* (Hance) Baker			
*Iris dichotoma* Pall. ^1^			**3.34 ^4^**
SS03-1	Cheonripo Arboretum (regenerated individual from a wild population); SS	32	3.35 ± 0.004
SS20-1	Cheonripo Arboretum (regenerated individual from a wild population); SS	32	3.35 ± 0.008
SS20-2	Cheonripo Arboretum (regenerated individual from a wild population); SS	32	3.32 ± 0.011
SS20-3	Cheonripo Arboretum (regenerated individual from a wild population); SS	32	3.34 ± 0.006
*I. domestica* (L.) Goldblatt & Mabb.			
Beom_1	Busan (cult.); CB, YK	32	4.75 ± 0.010
*Sisyrinchium rosulatum* E.P.Bicknell.			**0.91 ^4^**
JCKC190515	Hyomyeong, Jeju; N33°19′25″, E126°35′39″, 327 m; TS, CB, SK, CM	32	0.91 ± 0.001
JCKC190451	Gujwa, Jeju; N33°27′05″, E126°48′06″, 244 m; TS, CB, SK, CM	32	0.90 ± 0.001

^1^ GPS coordinates, latitude and longitude of the collected sites of endangered, threatened and endemic species are not indicated for protection purpose (collected under permits no. 2018-18, 2019-13, 2019-14, 2019-20, and 2019-30 from the Ko-Seong, Dae-Gu, Dae-Jeon, Busan, and Jeon-Nam populations, respectively); ^2^ Taxa endemic to Korea; ^3^ Taxa subendemic to Korea; Collectors: TS = Tae-Soo Jang; CB = Bokyung Choi; KS = Sun-Yu Kim; YK = Yusun Kweon; RI = Il Roh; SK = Seong-Yeon Kang; SS = Soonku So; CM = Young-Min Choi; YC = Yeongmun Choi; YS = Sungyu Yang; SJH = Jun-Ho Song; ^4^ Results in bold indicates mean genome size values for each species.

**Table 2:** Karyotype analyses in the genus *Iris* L. and its related taxon *Sisyrinchium rosulatum*.

Taxon (Collection Number)	Chromosome Length (µm)			AsI ^2^ (%)	RI ^3^	Figure
	Largest (Mean ± S.D.)	Smallest (Mean ± S.D.)	Total HKL ^1^ (Mean ± S.D.)			
Iris						
Subgenus *Limniris* section *Limniris*						
*Iris ensata* (IMHAE117)	4.8 ± 0.2	2.8 ± 0.1	82.5 ± 1.7	66.7	1.69	1A, 2A
*I. koreana* (sck00043)	5.3 ± 0.8	1.5 ± 0.1	141.7 ± 9.9	61.9	3.51	1B, 2B
*I. laevigata* (Jebi-2)	3.7 ± 0.1	2.0 ± 0.1	81.8 ± 1.7	61.9	1.85	1 C, 2C
*I. minutoaurea* (Cheonma-2)	5.1 ± 0.2	1.9 ± 0.1	75.8 ± 2.4	58.3	2.58	1D, 2D
*I. odaesanensis* (YSG18-66_2)	4.3 ± 0.1	1.9 ± 0.1	86.3 ± 2.4	59.5	2.19	1E, 2E
*I. oxypetala* (CC0621-50)	2.9 ± 0.1	1.7 ± 0.1	86.4 ± 3.8	59.1	1.72	1F, 2F
*I. pseudacorus* (CC20190621-57)	4.9 ± 0.3	2.1 ± 0.1	107.1 ± 4.2	62.0	2.34	1G, 2G
*I. rossii* (Keumjeong_8)	4.5 ± 0.2	2.1 ± 0.1	100.5 ± 3.0	60.9	2.08	1H, 2H
*I. sanguinea* (Hwaya_1)	4.5 ± 0.2	2.1 ± 0.2	87.2 ± 2.8	62.5	2.09	1I, 2I
I. setosa (Seo-2)	4.6 ± 0.4	2.1 ± 0.1	122.9 ± 6.9	61.6	2.11	1J, 2J
Subgenus Limniris section Ioniris						
*I. ruthenica* (JC534-1)	2.9 ± 0.2	1.1 ± 0.1	78.3 ± 4.3	58.0	2.52	1K, 2K
*I. uniflora* (JCK2019-79)	2.3 ± 0.2	1.1 ± 0.1	61.9 ± 1.9	58.3	1.95	1L, 2L
Subgenus Pardanthopsis						
*I. dichotoma* (SS20-3)	4.6 ± 0.3	2.0 ± 0.1	97.0 ± 4.4	61.9	2.19	1M, 2M
*I. domestica* (Beom_1)	3.9 ± 0.3	2.1 ± 0.1	92.8 ± 4.2	59.7	1.76	1N, 2N
*Sisyrinchium rosulatum* (JCKC190515)	1.8 ± 0.1	1.0 ± 0.1	43.4 ± 1.9	59.9	1.84	1O, 2O

^1^ HKL: total haploid karyotype length; ^2^ AsI (asymmetry index): the proportion of all long arms to the total haploid karyotype length; ^3^ RI (Ratio Index): the ratio of the longest to the shortest chromosome.

## Supplementary Materials

The following are available online at https://www.mdpi.com/2223-7747/9/10/1284/s1, Table S1. Chromosome counts from the published data according to Rice et al. [17].
